# WearETE: A Scalable Wearable E-Textile Triboelectric Energy Harvesting System for Human Motion Scavenging

**DOI:** 10.3390/s17112649

**Published:** 2017-11-17

**Authors:** Xian Li, Ye Sun

**Affiliations:** Department of Mechanical Engineering-Engineering Mechanics, Michigan Technological University, 1400 Townsend Drive, Houghton, MI 49931, USA; xian@mtu.edu

**Keywords:** triboelectric energy harvesting, wearable electronics, human motion

## Abstract

In this paper, we report the design, experimental validation and application of a scalable, wearable e-textile triboelectric energy harvesting (WearETE) system for scavenging energy from activities of daily living. The WearETE system features ultra-low-cost material and manufacturing methods, high accessibility, and high feasibility for powering wearable sensors and electronics. The foam and e-textile are used as the two active tribomaterials for energy harvester design with the consideration of flexibility and wearability. A calibration platform is also developed to quantify the input mechanical power and power efficiency. The performance of the WearETE system for human motion scavenging is validated and calibrated through experiments. The results show that the wearable triboelectric energy harvester can generate over 70 V output voltage which is capable of powering over 52 LEDs simultaneously with a 9 × 9 cm^2^ area. A larger version is able to lighten 190 LEDs during contact-separation process. The WearETE system can generate a maximum power of 4.8113 mW from hand clapping movements under the frequency of 4 Hz. The average power efficiency can be up to 24.94%. The output power harvested by the WearETE system during slow walking is 7.5248 µW. The results show the possibility of powering wearable electronics during human motion.

## 1. Introduction

Wearable sensors provide a sustainable solution for physical and physiological monitoring and well-being management. Energy harvesting technologies have been widely considered to prolong the lifetime of wearable devices by extracting energy from ambient resources. Among them, human motion is one of the most significant energy resources that can be harvested to power wearable sensors and electronics, enabling self-sustainability [1]. Generally, three mechanisms are commonly used to convert mechanical energy to electricity for sensors: piezoelectricity based on piezo materials [2,3,4,5,6], electromagnetics based on Lenz’s Law [7,8,9] and electrostatics based on variable capacitance [10]. For example, Aidin et al. [2] developed a flexible piezoelectric generator to harvest energy from ear canal motion. Pillatsch et al. [11] tested and built a piezoelectric rotational energy harvester, which can harvest peak power of 7 µW while mounting the device on an upper arm during running.

Triboelectric energy harvesters are a new type of energy harvesting technique since the first one developed in 2012 [12], which is based on the mechanism of triboelectricity. Contact and separation between two materials can generally generate charges, which provides an alternative mechanism for converting mechanical energy to electricity power. These harvesters are able to generate a high voltage peak (V_pp_ > 20 V [13]) at random and low frequency. A few remarkable devices have then been demonstrated to harvest human motion and power wearable electronics with harvesters embedded on shoes [14] or attached to the cloth [15]. Bai et al. [16] demonstrated a triboelectric harvester which can generate an instantaneous maximum power density of 9.8 mW/cm^2^ and also can drive multiple LEDs with the similar device. Yang et al. [17] proposed a triboelectric nanogenerator which can produce power with a peak power density of 30.7 W/m^2^. They also mounted the harvester to a backpack to harvest energy, which can light up to 40 LEDs instantaneously, from human walking. Their harvesters, so-called triboelectric nanogenerator (TENG), are remarkable based on nanotechnology with various surface structures.

Considering the cost, scalability, and wearability for daily use, we propose a lightweight, ultra-low-cost wearable triboelectric harvesting system, namely WearETE, based on e-textile and foam for daily human motion harvesting. We prove the concept that flexible and low-cost manufacturing methods and common materials can also produce effective triboelectric energy harvesting performance. Due to its flexible and scalable features, the WearETE system is able to be mounted with cloth or shoes or carried in the pocket to harvest energy from human motion, which provides the feasibility for powering wearable sensors and electronics. This paper reports the system design, experimental calibration and validation, and daily application of the scalable and wearable WearETE system. A testing platform which is capable of measuring the acceleration and external force is also proposed and developed to quantify the input mechanical power and power efficiency, which can be used as a calibration platform for triboelectric energy harvesting calibration.

This paper is organized as follows: Section 2 presents the mechanism and the design of the WearETE system and the system performance calibration method. The validation and calibration experiments and the daily application of the WearETE system are presented in Section 3, followed by the conclusion in Section 4.

## 2. Materials and Methods

**Figure 1:**
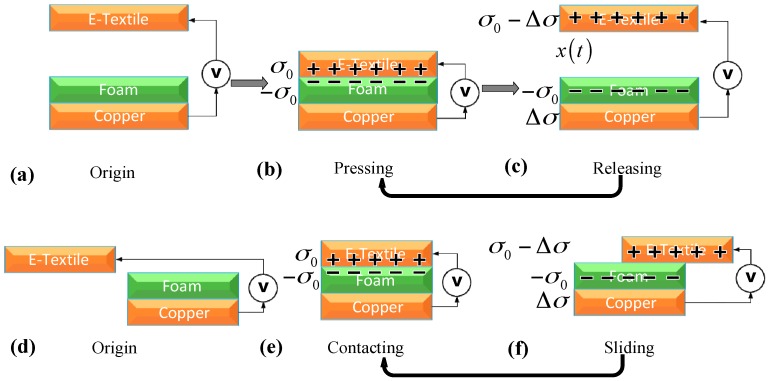
Mechanism of triboelectric energy harvester (pressing and sliding models). For pressing model, (**a**) original position; (**b**) pressing; (**c**) releasing. For sliding model, (**d**) original position; (**e**) contacting; (**f**) sliding.

Triboelectricity is a well-known phenomenon and often considered as harmful because it may cause damages to industrial electronics and non-comfort to human. Triboelectric energy harvesters use this harmful phenomenon to generate electrical power. Contact and separation, rubbing and friction between two different or even seemingly chemically identical materials, often result in electrical charge generation and distribution with opposite signs on either surface. This ubiquitous phenomenon is often referred to as triboelectricity when the contact materials are both solids. Electron transfer theory is generally believed as the mechanism for triboelectric charge generation [18,19]. Contacting and materials have been proved to be major factors that cause charge generation [20,21]. The relative movement of the two tribomaterials can be longitudinal or transverse corresponding to pressing and sliding as shown in Figure 1. In this section, Section 2.1 describes the motion conversion mechanism. Section 2.2 and Section 2.3 present the tribomaterial selection and the design and fabrication, respectively. Section 2.4 provides an overview of the WearETE system. Section 2.5 introduces the newly proposed platform to measure and calibrate input power and power efficiency.

### 2.1. Mechanism of Triboelectric Energy Conversion

The energy conversion of the proposed energy harvester is based on triboelectric charge generation and redistribution caused by contact and relative motion of the two tribomaterials. The generated charges can then be collected for power generation. The relative motion of the two tribomaterials can be longitudinal or transverse corresponding to pressing and sliding during human motion.

With regard to real system design for mechanical energy conversion, the principle of inertia is generally used that a frame with a movable mass is attached to a mechanically moving source and the relative motion is then controlled by the law of inertia [22,23]. The system is made resonant to generate the contact-separation motion of the two tribomaterials by suspending the movable part to a spring. The system forms a unified mass-spring-damper system. A portion of the kinetic energy of the moving mass is converted into electrical energy; whereas some is damped by parasitic effects of the system. A basic model of the mass-spring-damper system with the consideration of mechanical energy conversion is shown in Equation (1).

(1) $$m\ddot{x}+(c_{elec}+c_{mec})\dot{x}+kx=-m\ddot{y}$$

where x represents the motion of the mass; $c_{elec}$ and $c_{mec}$ are the damping coefficients caused by the mechanical-to-electricity conversion and the mechanical damping effects, respectively; y is the frame movement; k is the spring constant. In this case, we assume that the frame of the mass-spring-damper system moves simultaneously with the external force. In the triboelectric energy harvester experiment, there are two possible conditions. One can be modeled by Equation (1) that the bottom part, which serves as the reference, is fixed in the experiment, the analytic solution for free vibration causing contact-separation of the two tribomaterials is described as Equation (2),

(2) $$x\left(t\right)=X_{0}e^{-\zeta \omega_{n}t}\cos\left(\sqrt{1-\zeta^{2}}\omega_{n}t-\phi_{1}\right)$$

where x(t) is the displacement of the mass relative to its equilibrium position; ζ and $\omega_{n}$ are the damping ratio and the natural frequency of the object, respectively, where $\zeta =\left(c_{elec}+c_{mec}\right)/2\sqrt{mk}$ refers to the entire damping; t is the time; and $\phi_{1}$ is the phase shift. The other condition is to generate contact-separation with measurable motion without using mass-spring-damper system. In this condition, x(t) is controlled by the hand clapping movements in the experiment.

In the WearETE device, the area of layers is considered as infinite comparing to the small separation distance. Thus, the generated potential on the electrode can be presented as Equation (3),

(3) $$U_{tribo}=\sum_{i=1}^{N}\sum_{j=1}^{M}E_{ij}X_{j}$$

where $E_{ij}$ is the electrical field generated by the surface charges due to contact-separation, $X_{j}$ is the thickness or distance of layer j. Therefore, the generated potential can be further calculated by Equation (4),

(4) $$U_{tribo}(t)=\frac{\sigma }{\varepsilon_{0}}x\left(t\right)$$

where σ is the surface charge density generated by contact which is also related to the material property;$\varepsilon_{0}$ is the vacuum permittivity.

The mechanism of charge generation in our proposed WearETE is shown in Figure 1. The e-textile and foam layers are flexible and can generate charges by both contact-separation and sliding. The contact-separate-contact process is shown in Figure 1a–c. The mechanism of sliding is similar to contact-separation as illustrated in Figure 1d–f.

### 2.2. Material Property and Selection

Various materials can have triboelectric effects. The polarization ability of the two contact materials influences the performance, which forms the well-known triboelectric series [24,25]. When two tribomaterials contact or slide with each other, there will be charges on the surfaces with opposite sign. The material on the higher end of the triboelectric series is easier to lose electrons during contacting or sliding. The two materials far from each other in the series often result in higher voltage. Charge affinity is used by some studies to characterize this polarization property [26]. Thus, the triboelectric series can be used as the reference for material selection.

Two different materials far from each other in the triboelectric series are selected to develop the WearETE. One principle for choosing these two materials is their different polarization abilities. The two tribomaterial layers need to be relatively easy to lose and obtain electrons, respectively. In addition—due to the goal of wearability—flexibility and stretchability are also considered. Therefore, we first tested the performance of the common textile materials in the triboelectric series. An experiment was conducted in the study to investigate the triboelectric property of flexible textile materials, including cotton, polyester, paper, and foam. The experiment and results have been presented in Section 3.1. In the results, foam shows high charge generation capability when sliding with the conductive copper substrate. For developing a wearable system, e-textile is often used as the wearable conductive material. Surprisingly, it shows high charge generation when contacting with foam. E-textiles, also known as electronic textiles, are fabrics with embedded conductive threads or electronic components to enable electric performance and have been used in wearable sensing and smart clothing instead of traditional conductors. In e-textile, stainless steel fibers are generally woven or knitted into cloth to enable conductivity. In our system design, the e-textile layer is used as both a tribomaterial layer and an electrode. Due to the high effectiveness and low cost, e-textile and foam are chosen as the two materials of WearETE in the system design.

### 2.3. Design and Fabrication of Energy Harvester

**Figure 2:**
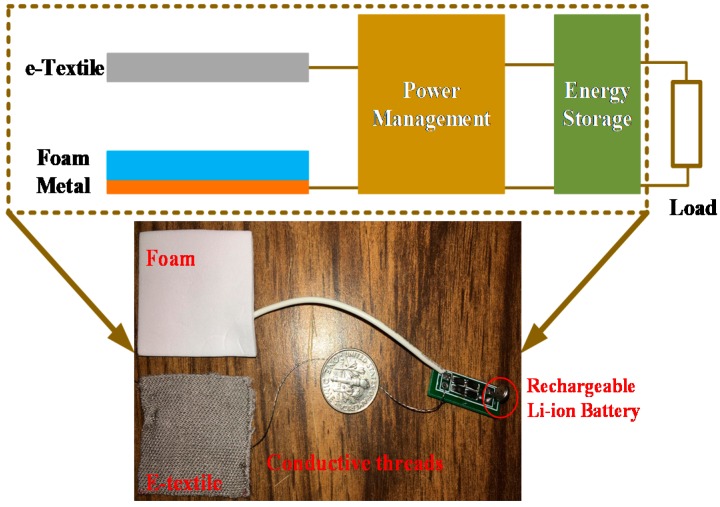
System architecture.

The triboelectric energy harvester consists of three major components: substrates for motion conversion, two tribomaterial layers for charge generation and two electrodes for charge collection. The substrates acting as supporting materials for motion conversion can be selected based on application scenarios. The substrates are selected to be solid for system calibration (e.g., 3D printed ABS boards) and flexible in real application (e.g., latex rubber). In addition to the two tribomaterial layers, electrodes are also needed to form a changeable capacitance. The e-textile layer, because of its conductivity, is used as both a tribomaterial layer and an electrode. A copper layer is adopted as another electrode. The two electrodes are then attached to the substrates which support and harvest mechanical motion. The fabrication of the entire system is flexible, scalable, and very low-cost. The design is illustrated in Figure 2.

### 2.4. WearETE System

The WearETE system is composed of three major components including the triboelectric energy harvester, the power management circuit, and an energy storage component. The conductive stainless-steel thread is used to connect different parts for wearability. The entire system architecture and demonstration is shown in Figure 2. The power management circuit is a simple bridge rectifier to demonstrate the feasibility. The AC power generated by the motion via the triboelectric energy harvester is converted into DC to power electric devices, such as LEDs, capacitors and cell batteries. In order to store harvested energy, capacitors or small li-ion batteries can be used as energy storage components.

### 2.5. Power Efficiency Estimation

**Figure 3:**
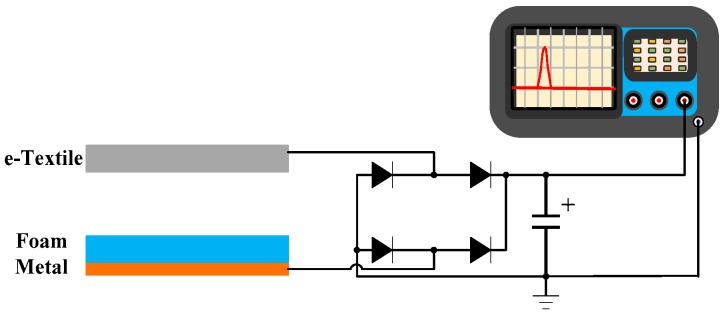
System setup with rectifier and display.

Due to the randomness and irregularity of human motion, the measurement of input mechanical energy and power efficiency of human motion energy harvesters calls for a standard method for system performance calibration and evaluation. In this study, we propose a new measurement platform which can be used for calibrating random human motion energy conversion. In order to quantitatively estimate the power efficiency generated from low-frequency motion, a testing platform and the associated calculation methods are established. The power efficiency refers to the ratio of the generated electrical power to input mechanical power. Usually, for piezoelectric energy harvesting, the maximum power can be harvested at the resonant frequency. However, because of the low frequency and irregular nature of human motion [27,28], the triboelectric energy harvester may not be able to achieve the resonant frequency. In this study, we focus on the performance of the proposed system in low frequency range (<5 Hz). The experimental setup is shown in Figure 3. A capacitor is used to estimate the generated power, which can be calculated by Equation (5) [29]

(5) $$P_{out}(t)=\frac{dW}{dt}=C\cdot V(t)\cdot \frac{dV(t)}{dt}$$

where V(t) is the voltage of the capacitor, C, during energy harvesting and can be measured by an oscilloscope; $P_{out}(t)$ is the generated power that stored in the capacitor at time t. The maximum power, $P_{\max}$**,** can be found by Equation (5) as well [29]. Then the generated average power $P_{avg}(t)$ during the time period $t_{i}$ to $t_{j}$ can be calculated as

(6) $$P_{avg}=\frac{1}{t_{i}-t_{j}}\int_{t_{i}}^{t_{j}}P_{out}\left(t\right)\cdot dt$$

Human motion has its nature of randomness and irregularity and therefore is challenging to calibrate as a standard mechanical input. Thus, we are using an average value in a time duration to represent the generated average electrical energy. The equation used to calculate average power (or effective power) in Reference [30,31,32] is $I^{2}R$, which is normally adopted for calculating power based on the measured current I and the known resistance R. In our case, we use a load capacitor to calibrate the output energy, which is an alternative method and widely used to calibrate output energy of energy harvesters such as [33,34,35]. As the goal of our study is to harvest the energy from human motion (e.g., the hand clapping movements) and then store, the capacitors are suitable for storing the harvested energy as well.

**Figure 4:**
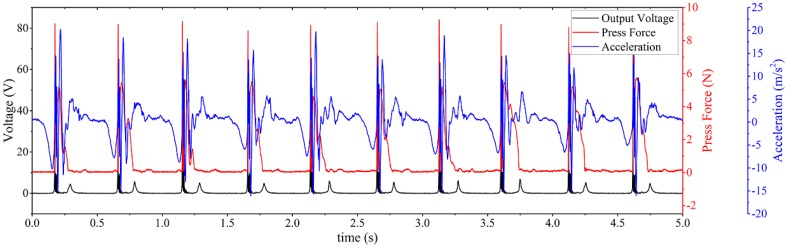
Typical synchronized measured signals during tapping including voltage output of energy harvester, force sensor and accelerometer.

To estimate the power efficiency for kinetic energy harvesting, a known vibration input is generally adopted. However, there is no standard method to evaluate energy harvesting from human motion. In this study, we propose a measurement platform to quantitatively estimate the input power of human motion. Three signals are needed to calibrate the input human motion, including acceleration, velocity, and external force. The acceleration signal is obtained via a 3-axis accelerometer. The velocity can be then obtained via integration of acceleration. The external force signal is measured by a force sensitive resistor sensor (FlexiForce A201, Tekscan, Inc., Boston, MA, USA). There is a linear relationship between the external force applied to the sensor and the sensor resistance. This linearity can be obtained from the calibration data. A simple voltage divider is also applied to measure the voltage of the force sensor and then calculate the external force. For the measurement of acceleration, we used an accelerometer from PCB Piezoelectronics, Inc. (Depew, NY, USA) (model 333B50) to measure in the input human motion. It has a sensitivity of (±10%) 102 mV/(m/s²) with ultrahigh linearity, which is highly suitable for the human hand clapping movements. The voltage outputs of the accelerometer and the force sensor have been measured using an oscilloscope (PicoScope 4424, Pico Technology, Cambridgeshire, UK). The probes we used in the measurements are high impedance passive probes. The transferred data are analyzed offline. The typical synchronized signals are shown in Figure 4, which corresponds to the motion process. In Figure 4, it is shown that the force signal during the intervals became a very small value, which means the two substrates of the triboelectric energy harvester were nearly separated at that moment. Comparing with the output voltage of the WearETE system, it shows that the second peak of the output voltage comes from the separation motion. Small output voltage of WearETE at that moment may result from moment of inertia.

**Figure 5:**
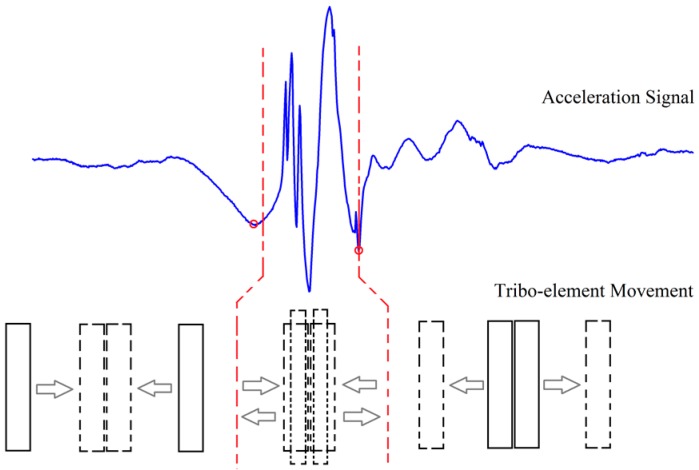
Schematic of the three sessions during tapping.

In the detected signals, multiple typical points are shown in the acceleration signal to divide the entire motion process into three sessions, which aligns with the real motion. The schematic of the three sessions has been shown in Figure 5. In the first session ($t_{0}$ to $t_{1}$), the two substrates of WearETE move from their original points (i.e., maximum separation distance) to the point where they contact each other. In the second session ($t_{1}$ to $t_{2}$), the two tribomaterial layers keep contacting. Due to moment of inertia, these two substrates keep moving for a short distance (less than their thickness) in the first half and then move in the opposite direction till the moment of separation in the second half. In the last one ($t_{2}$ to $t_{3}$), they move back to the maximum separation distance. Finally, the two substrates are back to the original places as one period. Thus, the input power in the first and third sessions can be calculated from the acceleration signal as shown in Figure 4 using Equation (7); whereas that in the second session can be calculated from both the force sensor signal and the acceleration signal using Equation (8).

(7) $$P_{in1}\left(t\right)=m_{1}\cdot v\left(t\right)\cdot a\left(t\right)$$

(8) $$P_{in2}\left(t\right)=m_{2}\left(t\right)\cdot g\cdot v\left(t\right)$$

where $m_{1}$ is the mass of one half of the triboelectric energy harvester layer; $m_{2}\left(t\right)$ is the measured external force during contacting; v(t) and a(t) are the velocity and acceleration during motion; g is the gravity constant. Then the input power can be calculated by a piecewise function as Equation (9),

(9) $$P_{in}\left(t\right)=\{\begin{array}{c} m_{1}\cdot v\left(t\right)\cdot a\left(t\right),\;\mathrm{for}\;t_{0}\leq t\leq t_{1}\;\mathrm{or}\;t_{2}\leq t\leq t_{3}\\ m_{2}\left(t\right)\cdot g\cdot v\left(t\right),\;\mathrm{for}\;t_{1}<t<t_{2} \end{array}$$

The power efficiency for WearETE can be calculated by Equation (10) within synchronized time duration for input power, $P_{in}$ and output power, $P_{out}$.

(10) $$\eta \;=\;\frac{P_{out}}{P_{in}}$$

## 3. Experiment and Results

### 3.1. Energy Harvester Validation

**Figure 6:**
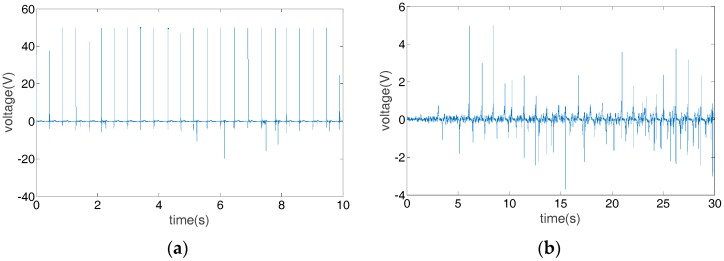
Performance of the harvester during (**a**) contact-separation motion and (**b**) sliding motion.

**Figure 7:**
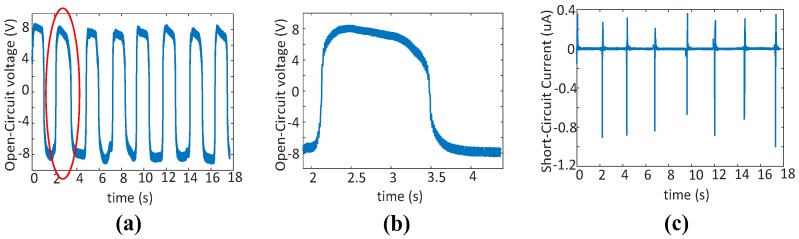
Open-circuit voltage and short-circuit current of the triboelectric energy harvester (PTFE and copper, pressing model). (**a**) The open-circuit voltage; (**b**) the enlarged figure of one period in (**a**); (**c**) short-circuit current.

In the energy harvester validation experiment, we first test the open-circuit voltage and short-circuit current to calibrate the energy harvester performance and then measure the output on a regular oscilloscope without rectifier to compare with the cases with load capacitors for calibration. The oscilloscope used for testing the open-circuit voltage and short-circuit current in this study is an Electrochemistry Workstation (model: CHI660B, CHI Instruments, Inc., Austin, TX, USA). The typical input impedance of reference electrode is 10^12^ Ω. The probes for measurements are high performance passive probes with high impedance. The oscilloscope for system calibration with load capacitors is a regular digital oscilloscope (PicoScope 4424, Pico Technology, Cambridgeshire, UK). Triboelectric energy harvester can have two modes for harvesting human motion as shown in Figure 1, the contact-separation mode and the sliding mode. The voltage outputs without the rectifier are shown in Figure 6. For contact-separation motion, the voltage output is more stable and has a larger magnitude than that during sliding motion. For sliding motion, due to its irregular nature, the voltage output is unstable but it still generates considerable power. These results indicate that triboelectric energy harvester can harvest energy from both types of motions. An experiment has been conducted to test the open-circuit voltage and short-circuit current of the contact-separation mode. With the distance of the two layers, i.e., e-textile and foam, increasing until the maximum separation, the open-circuit voltage rises towards a maximum value following Equation (4). The pressing process is opposite. In a short-circuit condition, the resultant current appears as negative and positive pulses during each pressing and releasing cycle. The experimental results of the open-circuit voltage and short-circuit current as shown in Figure 7a–c align with the theoretical analysis.

**Figure 8:**
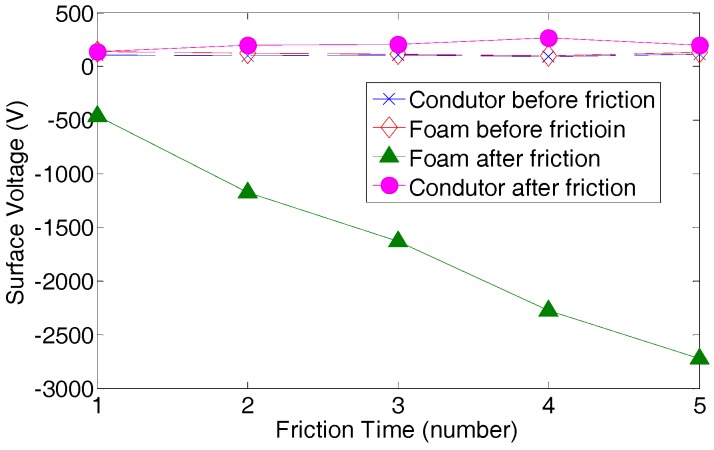
Triboelectric property of foam.

To select the two contact textile materials as discussed in Section 2.2, an experiment was first conducted in the study to investigate the triboelectric property of flexible textile materials including cotton, polyester, paper and foam. In the experiment, we prepared the samples of each material and slid them on the same copper substrate. A surface charge measurement device (USSVM2, AlphaLab Inc., Salt Lake City, UT, USA) was used to detect the surface voltage after each sliding. The charge accumulation can be clearly observed from the experiment that the surface voltage significantly increased with the sliding times. In the first experiment, we slid the four samples with copper substrate for five times and measured the surface voltage before and after sliding for all the four samples. For each sample, we tested at least five times. The average surface voltage caused by the generated triboelectric charges before and after sliding five times are listed in Table 1. Foam shows the highest charge generation capability when sliding with the conductive copper substrate, which is much higher than other materials. Then, we selected foam and measured the change generation of foam with sliding times. The surface voltage is almost linear with sliding times as shown in Figure 8. Although the exact value of the detected surface voltage varies, this result is repeatable in a certain range.

**Figure 9:**
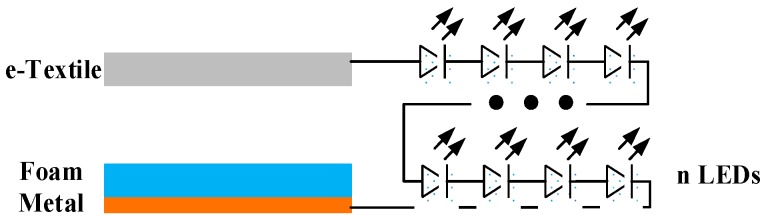
The Power Management Circuit for Powering a LED series.

**Figure 10:**
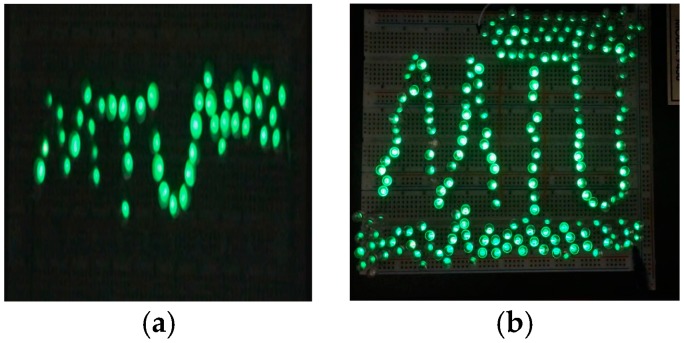
(**a**) A single WearETE powers 52 LEDs at each pulse. (**b**) A larger version of WearETE can power 190 LEDs.

In order to visualize the performance of WearETE, a number of LEDs were used to connect to the energy harvester as shown in Figure 9. In the experiment, two WearETE (9 × 9 cm^2^ and 14 × 21 cm^2^) were adopted to lighten 52 and 190 LEDs successfully, which have been shown in Figure 10.

### 3.2. System Performance

The WearETE system including the rectifier can harvest human motion to DC for powering purpose. Due to the irregular feature of human motion, it is challenging to control the input power as the same for all performed tests. In the experiment, we calibrate the system performance under different human motion conditions, which is similar to previous human motion energy harvester studies [27,28]. The energy harvesting in contact-separation motion mode is first validated. For energy harvesting, the capacitor and the exciting frequency govern the harvested energy. The capacitance generally influences the charging time constant and the output voltage. Thus, different capacitors under different frequencies are tested to calculate the power that the WearETE system can harvest and validate the system performance. The smaller the capacitance, the smaller the time constant, the faster it is charged or discharged and vice versa. The frequencies are chosen based on the nature of human motion which is less than 5 Hz. The power generated by WearETE during contact-separation mode can be calculated by Equation (5). The input mechanical energy is measured and calculated using the platform presented in Section 3. Then the power efficiency is calculated by Equation (10).

In the experiment, the accelerometer and the force sensor were precisely aligned to the backside of the WearETE during calibration, aiming to protect the triboelectric materials (e-texture, foam) from the impact caused by accelerometer and force sensor thin film. To acquire comparable results under different frequencies, the two substrates of the triboelectric energy harvester were pressed under repeated movement, which was validated using the force sensor. Under the stable tapping frequency, the output voltage of the WearETE system is periodic.

The typically measured output voltage of the WearETE system along with the calibration sensor data during contact-separation motion is shown in Figure 4. In this case, the motion frequency is 2 Hz. The results in this case show that the output peak voltage of the WearETE system with an area of 10.16 × 10.16 cm^2^ through contacting (first peak) and separation (second peak) are approximately 70 V and 5.5 V, respectively.

**Figure 11:**
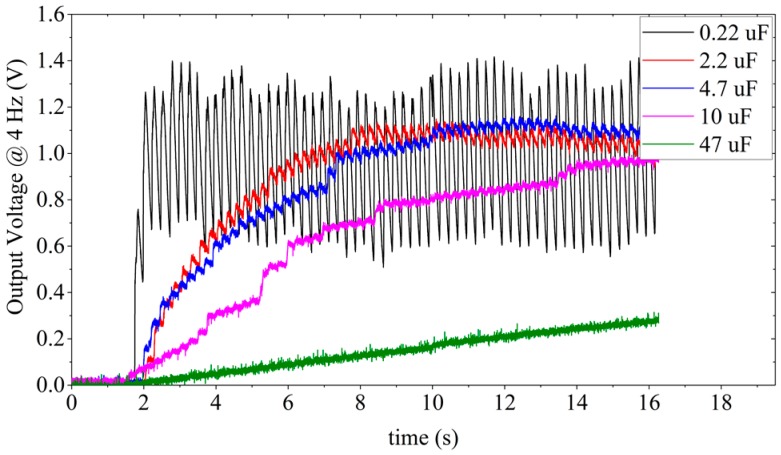
Charging different capacitors (0.22–47 µF) by WearETE under hand clapping movements with 4 Hz frequency.

**Figure 12:**
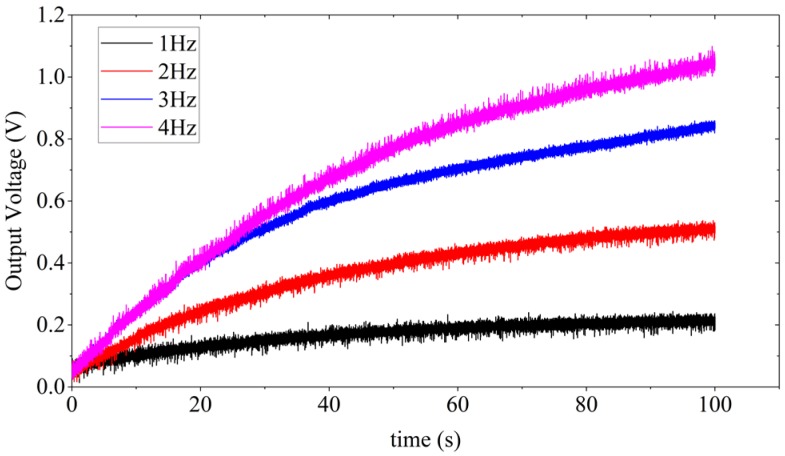
Charging 47 µF capacitor by triboelectric energy harvester under hand clapping movements with different frequencies (1–4 Hz).

In order to estimate the system performance of the WearETE during motion, a number of capacitors with different values, i.e., 0.22, 2.2, 4.7, 10 and 47 µF, are selected to test for charging under different frequencies, i.e., 1, 2, 3 and 4 Hz. The voltage through the capacitors during charging by WearETE system under 4 Hz are illustrated in Figure 11. The capacitor of 0.22 µF increases to 1 V most fast but it oscillates most dramatically. The capacitor of 47 µF increases slowly but more stable and discharges slowly as well. The results show that the voltage of the capacitors with larger capacitance increases slower and more stable and vice versa. The output voltage of the capacitor of 47 µF during charging under different frequencies are shown in Figure 12. The results show that the voltage of this large capacitor can be charged to 1 V in 100 s, which demonstrates the possibility in practical application. When the motion frequency is stable, the capacitor can be charged up to a stable value if the charging time is long enough.

**Figure 13:**
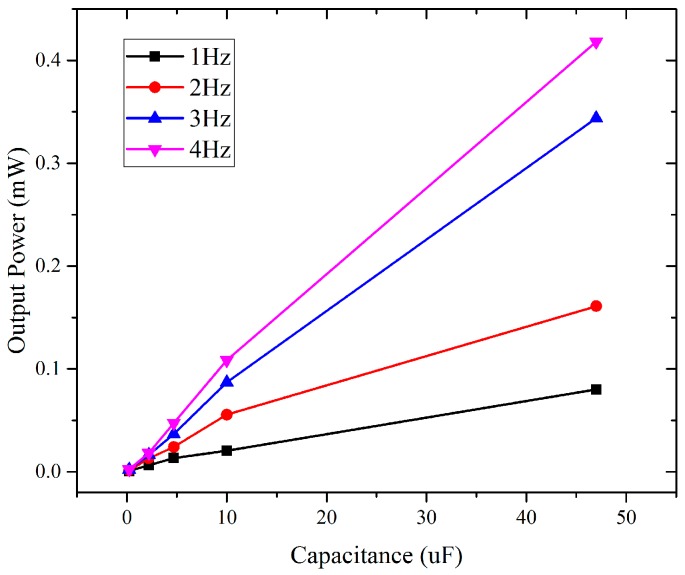
Average output power of charging different capacitors under hand clapping movements under different frequencies.

In addition, the power efficiency for charging different capacitors with different frequencies was also investigated in order to provide the estimation of the power generation due to frequencies of human motion and influence of the storage capacitors in the AC-DC process for entire energy harvesting system design. Figure 13 is to show how the output power changes with different input frequency due to human motion, which will provide an estimation of the power generation due to frequencies of human motion. The results show that higher load capacitance and input frequency generally cause higher output power. The results of charging a 47 µF capacitor under different motion frequencies are listed in Table 2. The input power is calibrated using the platform described in Section 2.5. In each experimental setting, i.e., different capacitances and frequencies, more than five sets of output voltage data were measured. These data show that the input power in different experiments are dispersed and varied in the range from 0.1 W to 1 W due to the irregular nature of human motion. Under the same experiment settings with similar level of force measured by the force sensor, the output power of larger capacitance or frequency during motion are much higher. It indicates that for charging a capacitor, higher frequency generates more energy in the same time interval. The conclusion is also applicable for charging a capacitor with higher capacitance. These trends are illustrated in Figure 13. The output power increases with the capacitance as well as the input mechanical frequency, which can provide the guidance in practical applications. Also, in the experiment, the power efficiency increased within 3 Hz; whereas the efficiency at 3 Hz is higher than that at 4 Hz due to the difference of the input power in these two cases. For charging a 47 µF capacitor, the power efficiency of average input and output power can be as high as 24.94%.

In addition to the average results, Table 3 summarizes the maximum power generated in these experimental settings with the capacitance of 0.22, 2.2, 4.7, 10, 47 µF under the frequency 1–4 Hz. From the experiment results, charging a 47 µF capacitor can harvest the highest maximum power, which is approximately 4.8113 mW, from the 4 Hz motion. The WearETE used in experiments has an area of 10.16 × 10.16 cm^2^, which equals to 103.226 cm^2^. The maximum power density thus can be 46.6 µW/cm^2^.

In the experiment, we found that the power loss caused by the bridge rectifier was high. In this study, since we focus on proposing and validating the proof-of-concept using low-cost materials and manufacturing method for power generation for wearable electronics, we will consider the power conditioning circuit design in the future work.

### 3.3. Harvesting Energy from Walking

During walking, people generally swing their hands to keep balance. Since the proposed WearETE system can generate power from sliding motion, a validation experiment was developed to harvest energy from people swinging their hands during walking. In the experiment, the two substrates of the WearETE were attached to the cloth on the side of body and front arm individually. Then the energy harvester was assembled with the power management circuit. The output voltage of the WearETE system is measured and recorded during walking. The power harvested during human walking is generated by sliding the two tribomaterials of the energy harvester. Finally, the output power is calculated based on the output voltage.

**Figure 14:**
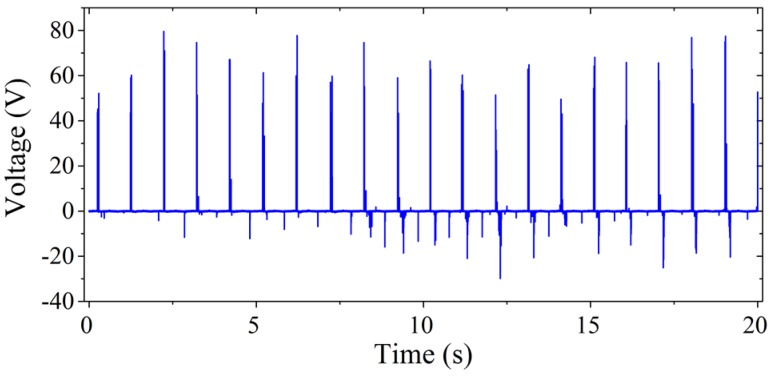
Output voltage of WearETE during harvesting energy from human walking.

The output of the WearETE system for harvesting energy from walking is shown in Figure 14. The output power harvested by WearETE during walking is 7.5248 µW. The results show the possibility for harvesting energy during human walking. Compared to other similar systems with energy harvester attached to the cloth [11], the WearETE system provides a reasonable performance for human motion energy harvesting. In this study, we demonstrate the feasibility of using very low-cost materials and fabrication methods for human motion harvesting.

## 4. Conclusions

In this study, we developed a novel scalable wearable e-textile triboelectric energy harvesting system for scavenging energy from daily human motion. The proposed energy harvester is scalable, stretchable, and wearable. The proposed WearETE system is based on triboelectric energy harvesting which is an emerging technique for kinetic energy conversion. The system adopts foam and e-textile as the tribomaterials, which demonstrates the feasibility of using low-cost materials and fabrication techniques for wearable energy harvester design. A platform for measuring and calculating power efficiency is also established for measuring and calibrating random human motion. The results show that the wearable triboelectric energy harvester can generate over 70 V output voltage which is capable of powering over 52 LEDs simultaneously with a 9 × 9 cm^2^ area. A larger version is able to lighten 190 LEDs during contact-separation process. The WearETE system can generate the maximum power up to approximately 4.8113 mW from human motion with the frequency of 4 Hz. The maximum area power density can be approximately 46.6 µW/cm^2^. The average output power harvested by WearETE system during slow walking is 7.5248 µW. The proposed WearETE system show the possibility of powering wearable electronics during human motion using a wearable energy harvester.

## Figures and Tables

**Table 1:** Surface voltage caused by triboelectric charge before and after sliding five times.

Material		Before Test (V)		After Test	
A	B	A	B	A	B
Polyester	Copper	98.9	112.2	51.5	125.0
Paper	Copper	130.3	113.2	539.5	−13.6
Cotton	Copper	124.9	116.3	355.3	169.0
Foam	Copper	123.6	137.9	−2725.6	201.6

**Table 2:** Average results of charging a 47 µF capacitor under hand clapping movements with different frequencies.

Frequency (Hz)	Input Power (mW)	Output Power (mW)	Efficiency (%)
1.0	3.6460	0.0802	2.1996
2.0	1.5351	0.1611	10.4918
3.0	1.3790	0.3439	24.9374
4.0	2.5519	0.4182	16.3891

**Table 3:** Maximum output power of charging different capacitors under hand clapping movements under different frequencies.

Maximum Power (mW)	1 Hz	2 Hz	3 Hz	4 Hz
0.22 µF	0.0222	0.0220	0.0265	0.0542
2.2 µF	0.0725	0.1182	0.1093	0.1700
4.7 µF	0.1656	0.1974	0.2402	0.4299
10 µF	0.1883	0.5115	0.5608	0.8986
47 µF	0.8288	1.6001	2.5916	4.8113
